# Iatrogenic Kaposi’s sarcoma in nasal cavity: a case report

**DOI:** 10.1186/1477-7819-12-172

**Published:** 2014-06-02

**Authors:** Kuang-Hua Chen, Tai-Di Chen, Chiang-Wen Chen, Li-yu Lee

**Affiliations:** 1 Department of Pathology, Chang Gung Memorial Hospital and Chang Gung University College of Medicine, 5 Fu Hsin Street, Kwei San, Taoyuan 333, Taiwan; 2 Department of Otolaryngology, Chang Gung Memorial Hospital and Chang Gung University College of Medicine, 5 Fu Hsin Street, Kwei San, Taoyuan 333, Taiwan

**Keywords:** Iatrogenic, Kaposi’s sarcoma, Nasal cavity, Systemic lupus erythematosus

## Abstract

**Background:**

Kaposi's sarcoma (KS) is an uncommon borderline vascular tumor involving mostly the cutaneous and mucosal sites of the body. Among the four distinctly clinicopathological presentations of KS, the iatrogenic form principally occurs in kidney transplant recipients receiving immunosuppressive therapy. It rarely occurs in the head and neck region as primary site or in other groups of patients under immunosuppressive therapy.

**Case presentation:**

We present of the case of a patient with right nose KS. The patient had history of systemic lupus erythematosus (SLE) and was under immunosuppressive therapy.

**Conclusion:**

Once we keep KS in mind, the definite diagnosis can be made using routine histological examination and immunohistochemical study despite the rarity of the disease in this site.

## Background

Kaposi's sarcoma (KS) was named after an Austrian dermatologist, Moritz Kaposi, who first described five cases of this unusual tumor in 1872 [1]. KS is primarily a borderline vascular tumor affecting the skin and mucosa, but in more advanced stages of the disease, the lymph nodes and almost any internal organ can also be involved. There are four distinctly clinicopathological presentations: the ‘classic’ or ‘sporadic’ form mostly occurring in the lower extremities in elderly patients with Mediterranean or Jewish ancestry, the rapidly progressive ‘epidemic’ form associated with the acquired immunodeficiency syndrome (AIDS), the ‘endemic’ form prevalent in middle-aged adults and children from sub-Saharan Africa, and the ‘iatrogenic’ form, which is associated with immunosuppressive therapies for organ transplants or other illnesses. Nonetheless, the histological features of all forms of KS are essentially the same, and human herpesvirus 8 (HHV8) is considered the causative agent for all forms of KS. Tumor development requires prior infection with HHV8 but HHV8 infection alone is not sufficient to induce it. Here, we present a patient with systemic lupus erythematosus (SLE) and an immunosuppressed state suffering from iatrogenic KS in the nose, a rarely primary site of KS.

## Case presentation

**Figure 1:**
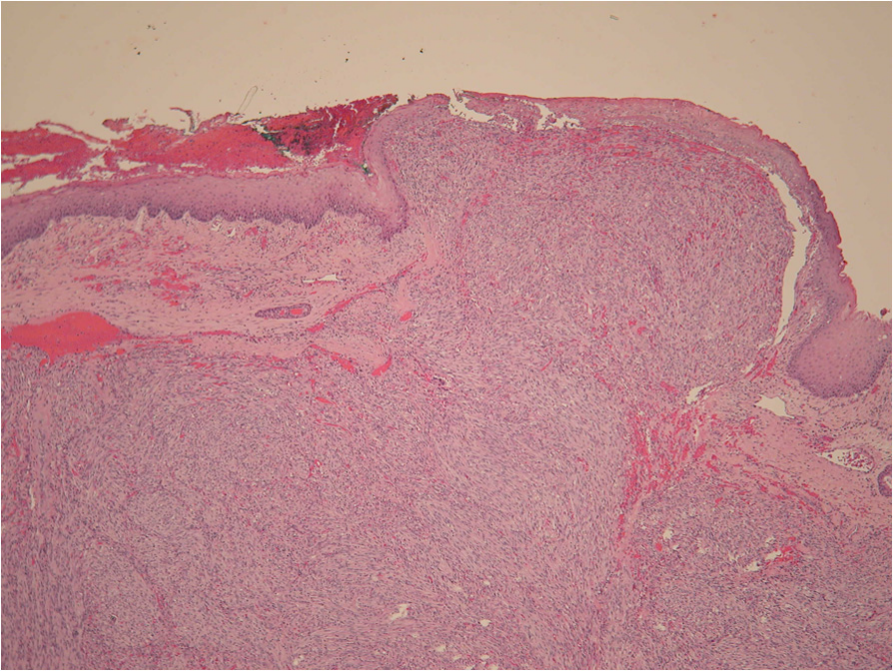
**Histopathology (40×, H & E staining).** Histological examination showed a polypoid submucosal tumor effacing normal structures.

**Figure 2:**
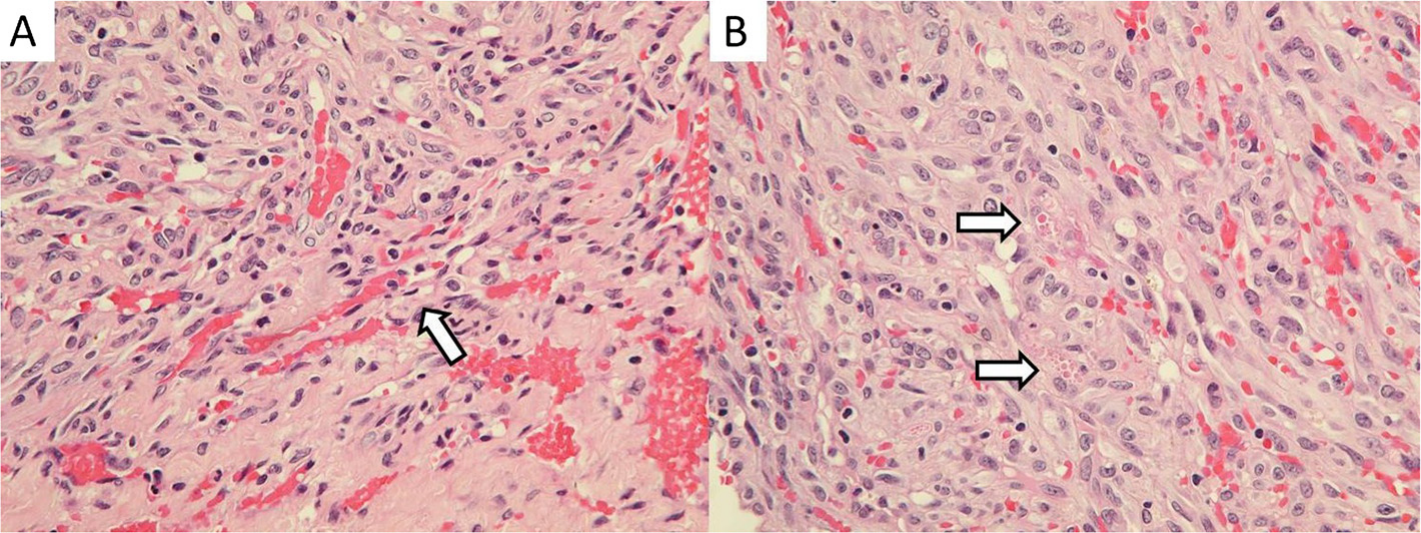
**Histopathology (400×, H & E staining). (A)** There are slit- and sieve-like vascular spaces formed by tumor cells with abundant erythrocytes. **(B)** Some eosinophilic hyaline globules were seen in tumor cells.

**Figure 3:**
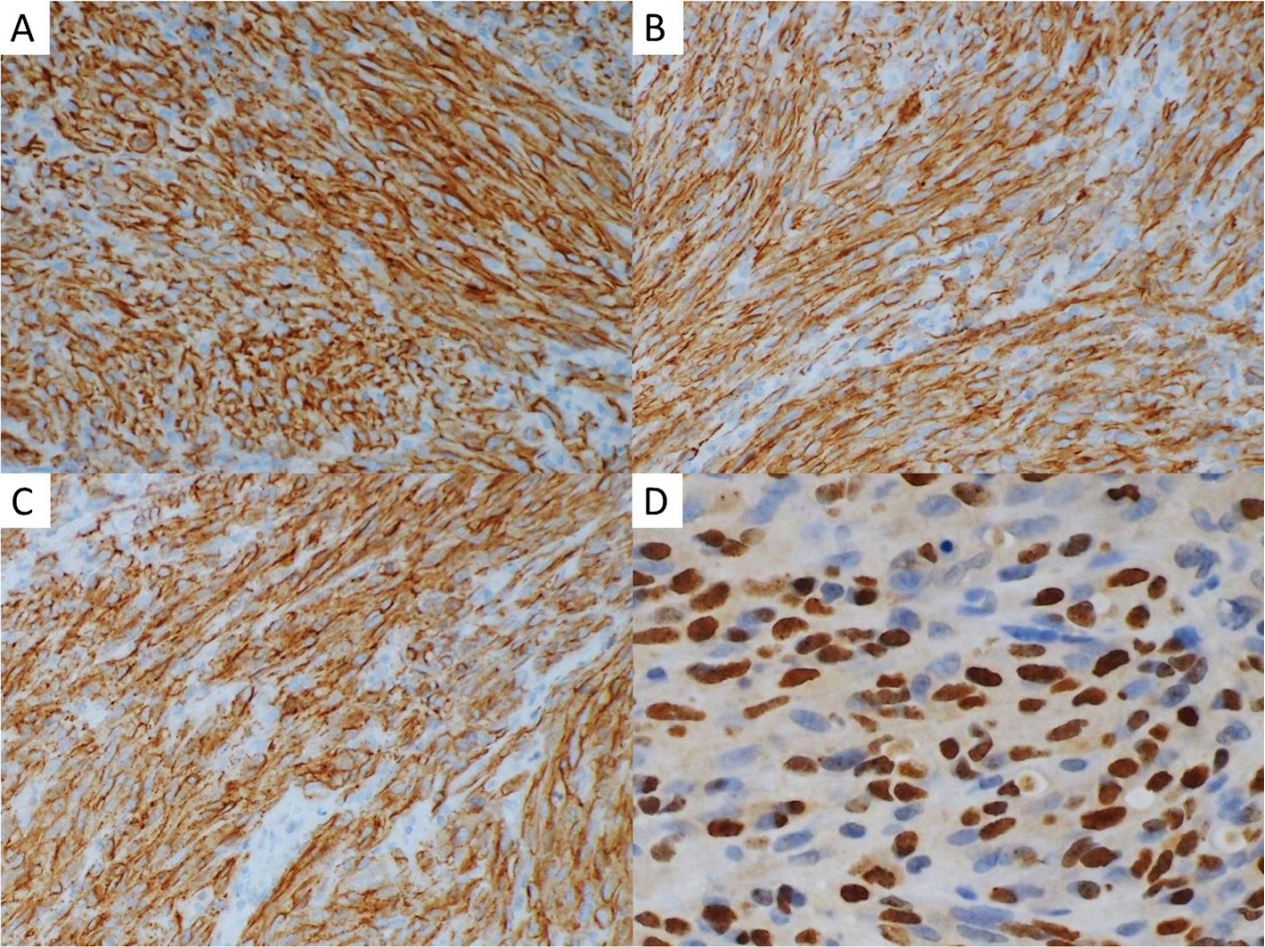
**Immunohistochemical stains.** The tumor cells were positive for CD31 (**A**, 200×), CD34 (**B**, 200×), and D2-40 (**C**, 200×) with nuclear stain for the latency-associated nuclear antigen (LANA-1) antibody (**D**, 400×). The diagnosis was confirmed.

A 31-year-old woman presented with a 1-month history of swelling on the right side of her nose. She also had a history of SLE with lupus nephritis and was under immunosuppressive therapy, mainly with prednisolone ranging from 10 to 20 mg per day for 9 years. A physical examination revealed no other specific findings. A sinoscopic examination showed a bulging nodule on her right anterior nasal floor near the nostril. Folliculitis with secondary infection was initially diagnosed, and amoxicillin with clavulanic acid was prescribed, but the lesion remained despite 1-week of the medication. A biopsy was undertaken for the suspicion of neoplasm.The specimen received consisted of five fragments of tissue measuring up to 0.5 × 0.4 × 0.1 cm with a soft consistency and focal hemorrhage. Histological analyses under hematoxylin and eosin (H & E) staining revealed a submucosal nodular tumor effacing normal structures (Figure 1). The tumor was composed of intersecting ill-defined fascicles of plump, spindled tumor cells with mild to moderate nuclear atypia. Mitoses were easily found. The tumor cells formed slit- and sieve-like vascular spaces containing erythrocytes (Figure 2A). Eosinophilic hyaline globules were seen in some tumor cells (Figure 2B). Inflammatory cells, mainly lymphocytes, and ectatic vessels were seen at the periphery of the tumor. Immunohistochemical (IHC) stains revealed that the tumor cells were strongly positive for CD31, CD34, D2-40, and latency-associated nuclear antigen (LANA-1) (Figure 3). Desmin, S-100, and AE1/AE3 immunostains were performed and the results were negative. Serological tests for human immunodeficiency virus antigen/antibody were negative. The final pathology diagnosis was iatrogenic Kaposi's sarcoma.

## Discussion

Iatrogenic KS principally occurs in kidney transplant recipients receiving immunosuppressive therapy. The incidence varies among different patient groups, but there is a 500- to 1000-fold difference compared to the general population [2]. The disease develops an average of 10 to 22 months after transplantation in this setting. The second group of patients at risk are those who received immunosuppressive therapy for a variety of medical reasons such as some hematological disorders, asthma, ulcerative colitis, or rheumatic diseases (such as SLE, as in our patient) [3]. In this group, KS develops around 41 months after the initiation of immunosuppressant therapy, and the incidence rate is 2- to 4-fold lower compared to renal transplant patients. The exact mechanism of KS associated with iatrogenic immunosuppression is unknown, but reactivation of the pre-existing HHV8 infection is thought to be the cause. Reactivation of the virus and the development of the KS are influenced by multiple factors including sex hormones, age, genetics, immune status, and cytokines [4]. Immunosuppression caused by steroid or other cytotoxic drugs could weaken the immunological surveillance mechanism. Additionally, some studies have shown that corticosteroids play a direct role in the development of Kaposi’s sarcoma, either by up-regulating KS cell proliferation or by reactivating the pre-existing HHV8 [5,6].

However, most patients receiving corticosteroids or other immunosuppressive regimens do not develop KS, especially in non-transplant settings. Although it has been stated that Kaposi's sarcoma also has a higher incidence in patients who have received immunosuppression therapy for various medical diseases, Louthrenoo *et al.* had found that only 24 documented rheumatologic patients from 1966 to 2002 developed KS [4]. Considering the worldwide extensive use of corticosteroids and/or other immunosuppressive drugs in patients with rheumatic disease, KS appears to be an extremely rare complication. There were only four cases of SLE complicated by KS documented in the English literature [4,7]. This finding supports the concept that the development of KS may require additional ethnic, genetic, or environmental predisposing factors other than immunosuppression alone. The rarity of this phenomenon could even raise the suspicion of a causal relationship between immunosuppression and the development of KS in these individuals. However, the regression of KS occurred in many of these patients after discontinuing or decreasing the dosage of immunosuppressive drugs, implying that the immunosuppressed state caused by these medications plays at least some role in the development of iatrogenic KS [4].

Although KS is well known to occur at almost any cutaneous and mucosal site of the body, the head and neck region is usually secondarily involved and rarely the site of initial presentation [8]. KS primarily involving the nasal mucosa is exceedingly rare, with only seven cases reported previously in the English literature; only two of them were not AIDS-associated presentations [9,10]. The nasal cavity is an infrequent location of primary malignant neoplasms. Approximately 1% of all human malignancies occur at this site, and more than 99% of these are squamous cell carcinoma [9]. The rarity of the otherwise not infrequently encountered Kaposi’s sarcoma in this location can become a pitfall in diagnosis. Clinically or pathologically, several diagnoses including lobular capillary hemangioma, spindle-cell hemangioendothelioma, spindle-cell angiosarcoma, glomangiopericytoma, bacillary angiomatosis, spindle-cell squamous cell carcinoma, and even synovial sarcoma could enter the differential list. However, if one keeps in mind the possibility of the existence of this tumor, it is often not difficult to establish or at least suspect the diagnosis of KS under routine H & E histological examination. The key point of correct diagnosis is in identifying the vessel-forming property of the tumor cells, the slit- and sieve-like vascular spaces, the spindle cells with relatively monotonous and low-grade nuclei, and the eosinophilic hyaline granules found in the cytoplasm. Additionally, CD31 (a pan-endothelial cell marker) and D2-40 (a lymphatic endothelial cell marker) are strongly expressed in both the spindle- and endothelial-cell components of Kaposi’s sarcoma. Finally, identifying the nuclear staining by immunohistochemical methods using the LANA-1 antibody essentially confirms the diagnosis.

The behavior and thus the treatment of KS depend on a number of factors such as the form of the disease, symptoms, location and extent of the lesion, the immunocompetence of the patient, and the general medical condition of the patient [9]. Local excision, radiation therapy, chemotherapy, and the adjustment of immunosuppressive medications could all be considered. Local recurrence is common in AIDS-KS patients but survival is more related to the immunological status of these patients; the mortality rate can reach 20 to 25% [10]. In patients with iatrogenic KS associated with immunosuppressants for rheumatic disease, tumor regression occurred in response to treatment in about two-thirds of cases [4]. Primary mucosal KS appears to have a similar prognosis to that of the typical cutaneous form presenting on the extremities [8]. Our patient had undergone local excision and low-dose radiation therapy (3000 cGy). The dosage of immunosuppressive medication was not adjusted. There was no recurrence during the four years of follow-up, prior to this case presentation.

## Conclusions

KS is an uncommon borderline vascular tumor, which occurs under various clinicopathological settings. KS is rare in the head and neck region and primary development in the nasal cavity is exceptional. Although iatrogenic KS is not uncommonly encountered in renal transplantation patients, it is rare in patients under immunosuppressive therapy for other medical reasons. The diagnosis of KS requires a high level of suspicion during histological examination and a panel of IHC studies would generally produce a firm conclusion. The treatment and prognosis depend on multiple factors and require consideration on a case-by-case basis.

## Consent

Written informed consent was obtained from the patient for publication of this case report and accompanying images. A copy of the written consent is available for review by the Editor-in-Chief of this journal.

## Abbreviations

AIDS: acquired immunodeficiency syndrome; H & E: hematoxylin- and eosin; HHV8: human herpesvirus 8; IHC: immunohistochemical; KS: Kaposi's sarcoma; LANA-1: latency-associated nuclear antigen; SLE: systemic lupus erythematosus.

## Competing interests

The authors declare that they have no competing interests.

## Authors’ contributions

CKH and CTD conceived the idea for the manuscript, conducted a literature search, and drafted the manuscript. CCW performed surgery and obtained images. LLY provided and reviewed pathological images, and critically revised the manuscript. All authors read and approved the final manuscript.
